# Mechanical countermeasures for spaceflight-associated neuro-ocular syndrome during 30-days of head down tilt bed rest: design, implementation, and tolerability

**DOI:** 10.3389/fphys.2025.1530783

**Published:** 2025-02-24

**Authors:** Stefan Moestl, Laura De Boni, Jan-Niklas Hoenemann, Tilmann Kramer, Jan Schmitz, Dominik Pesta, Timo Frett, Maria Bohmeier, Petra Frings-Meuthen, Ann Charlotte Ewald, Andrea Nitsche, Patricia Loehr, Alexandra Noppe, Nicolas Klischies, Alex S. Huang, Steven S. Laurie, Karina Marshall-Goebel, Brandon R. Macias, Jens Tank, Jens Jordan, Edwin Mulder

**Affiliations:** ^1^ German Aerospace Center (DLR), Institute of Aerospace Medicine, Cologne, Germany; ^2^ Department of Internal Medicine III, Division of Cardiology, Pneumology, Angiology, and Intensive Care, University Hospital Cologne, Cologne, Germany; ^3^ Department of Anesthesiology and Intensive Care, University Hospital Cologne, Cologne, Germany; ^4^ Centre for Endocrinology, Diabetes and Preventive Medicine (CEDP), University Hospital Cologne, Cologne, Germany; ^5^ Cologne Excellence Cluster on Cellular Stress Responses in Aging-Associated Diseases (CECAD), Cologne, Germany; ^6^ Medical Faculty, University of Cologne, Cologne, Germany; ^7^ UC San Diego Health, Shiley Eye Institute, San Diego, CA, United States; ^8^ KBR, Houston, TX, United States; ^9^ NASA Johnson Space Center, Houston, TX, United States

**Keywords:** SANS, bedrest, deconditioning, astronaut, microgravity, countermeasures, LBNP

## Abstract

After longer duration space missions, some astronauts experience structural and functional changes in the eye and structural changes in the brain, termed Spaceflight-Associated Neuro-Ocular Syndrome (SANS). Countermeasures against SANS are required to minimize potential operation impacts and negative long-term health consequences. Headward fluid shifts, which appear to promote SANS, provide a target for countermeasures. The SANS countermeasures study, a 30 days strict head down tilt bed rest (HDTBR) study, tested two mechanical countermeasures aimed at reversing cephalad fluid overload. This work presents design and methodology of the study with a focus on countermeasure implementation and tolerability. Following baseline evaluations, participants were randomized to four groups and HDTBR commenced: Daily application of 25 mmHg lower body negative pressure for 6 h, six-hour bilateral venous constrictive thigh cuffs following moderate cycling exercise on 6 days per week, a negative control group without countermeasures, and a positive control group with HDTBR interruption for 6 h per day by sitting upright. The potential of these countermeasures for future space applications was examined in 86 different experiments, which will be reported elsewhere. Comfort ratings ranging from 1 (very uncomfortable) to 5 (very comfortable) were used to asses tolerability. Overall, 47 participants (20 women) completed the study. Out of 4,032 h scheduled for both countermeasures, 10.5 h were not performed due to medical issues unrelated to the countermeasures. Mean comfort ratings were 3.9 in men and 4.4 in women in the lower body negative pressure group (p = 0.1356) and 4.2 in men and 3.9 in women in the thigh cuff group (p = 0.1604). We conclude that both countermeasures were well tolerated and applied under well controlled conditions, thus, allowing for meaningful analyses of efficacy in attenuating HDTBR effects.

## Introduction

During spaceflight, weightlessness causes multiple physiological changes in humans, which challenge performance and health during the mission and may not be fully reversible. In addition to mechanically unloading muscles, bones, the cardiovascular system, and the vestibular apparatus, weightlessness leads to a chronic redistribution of fluids throughout the body. Without diurnal changes in the hydrostatic fluid column, volumes and pressures in the head and neck are altered, affecting intravascular, interstitial, and possibly cerebrospinal fluid volumes and/or pressures. These changes are hypothesized to underlie the eye structural and functional changes observed in many astronauts and the resulting optic disc edema is the hallmark of the Spaceflight Associated Neuro-Ocular Syndrome (SANS) (Macias et al., 2020; Mader et al., 2011). Accordingly, countermeasures that reverse headward fluid shift hold promise in attenuating SANS. We have demonstrated that the strict head down tilt (HDT) bed rest model replicates SANS-like findings (Laurie et al., 2019; Laurie et al., 2020), but 30 min daily artificial gravity training via centrifugation was not sufficient to prevent optic disc edema (Laurie et al., 2021). These findings indicate that longer duration fluid shift countermeasures and upright-like hydrostatic pressure gradient may be needed to prevent SANS. The SANS countermeasures (SANS CM) study, which was jointly conducted by the National Aeronautics and Space Administration (NASA) and the German Aerospace Center (DLR), tested whether two mechanical countermeasures could reverse the headward fluid shift for several hours per day and successfully prevent SANS-like ocular changes during 30-days strict HDT bed rest. One countermeasure was lower body negative pressure (LBNP), which attenuates cephalad fluid shift by creating a pressure gradient redistributing fluids towards the lower extremities (Goswami et al., 2019). The other countermeasure, application of venous constrictive thigh cuffs, passively traps fluids in the lower extremities (Marshall-Goebel et al., 2021; Balasubramanian et al., 2018). To augment fluid accumulation, cuffs were donned after a moderate aerobic exercise, which has been shown to cause sustained systemic vasodilation (McCord et al., 2006). The study also included negative and positive control groups. The negative control group received no countermeasure and remained continuously in 6° HDT position. The positive control group interrupted strict HDT bed rest by sitting passively in a wheelchair. For future space missions, countermeasures must be safe and tolerable. For example, potential beneficial effects of LBNP training on orthostatic tolerance (Sun et al., 2002) have to be weighed against LBNP-induced presyncope, which could be deleterious in space (Ahmed et al., 2024). Therefore, in addition to the overall design and methodology of the SANS CM study, we report development and implementation of the countermeasures as well as their safety and tolerability.

## Methods

### Study participants

#### Ethical and regulatory approvals

Ethics committees in Germany (Ärztekammer Nordrhein, 2020211) and in the United States (IRB at Johnson Space Center, STUDY225 and STUDY235) approved the study. Approval to apply ionizing radiation was obtained from the German Federal Office for Radiation Protection (Bundesamt für Strahlenschutz, Z5-22464/2020-167-G). All participants gave their written informed consent and the study was prospectively registered in the German Clinical Trial Registry (DRKS00027643 for campaigns 1 + 2 and DRKS00030848 for campaigns 3 + 4).

#### Inclusion and exclusion criteria

Inclusion criteria included: Physically and mentally healthy test participants aged between 24 and 55 years; height between 153 and 190 cm; body mass index between 19 and 30 kg/m^2^; non-smoker status or abstinence from smoking for at least 6 months prior to the start of the study; possession of medical insurance; no criminal record; no more than 2 standard deviations below the average normal bone mineral density for hip and lumbar spine; demonstrable healthy dental certificate; no medication except hormonal contraception in women; and absence of any metallic implants that could interfere with MRI testing. In addition, participants had to confirm their availability for the study duration, including follow-up examinations, and their willingness to be assigned to any one of the four groups. Any medical condition precluding safe study participation (e.g., arterial hypertension, diabetes mellitus, thyroid dysfunction, migraine) led to the study exclusion. The fact that women on contraceptives were not excluded increased the number of female applicants compared to our previous joint DLR-NASA HDT bed rest studies called VaPER (Clément GR. et al., 2022) and AGBRESA (Clément G. et al., 2022).

#### Recruitment and medical screening

The call for applying to the SANS CM study started in September 2020 and was announced via social media and newspaper. Advertisements were also placed on the website of the Institute of Aerospace Medicine and on public digital displays. Eligible candidates in the DLR test participant registry were also contacted. Upon established contact, applicants had to fill out two general questionnaires, implemented as online surveys. The approach not only facilitated the application process for applicants, but also expedited evaluations by the recruitment team using partly computer-based questionnaire grading. For the next recruitment step, we provided a video containing all relevant information on scientific, medical, and organizational aspects of the study. This approach increased the number of applicants especially from remote areas. Furthermore, we were able to recruit during the COVID-19 pandemic without violating any legal or medical regulations. After watching the video, a checkbox appeared and applicants had to indicate whether they wanted to proceed with the application. However, as COVID-19 incidence surged, we had to postpone the study start from April 2021 to September 2021. Furthermore, we decided to only include persons who had been fully vaccinated against COVID-19. All suitable applicants then received our psychological questionnaires. After the questionnaires were evaluated by psychologists, applicants deemed suitable were scheduled for a telephone interview. In these interviews, we evaluated their proficiency in German, availability for the study duration, dietary considerations (e.g., habits, allergies, incompatibilities), and ensured they had a comprehensive understanding of the study protocol.

Following the interview, applicants underwent extensive medical screening at the DLR prior to enrollment (visit I). Screening comprised detailed medical history taking, complete physical examination, urine (e.g., nicotine/illicit drugs) and blood tests (e.g., clinical standard parameters for organ function, electrolytes, blood count, thrombophilia tests, pregnancy test for females), eye examinations, orthostatic tolerance testing (supine to standing), and electrocardiographic tests (resting and stress ECG). Eye disorder assessments encompassed visual acuity with and without corrective lenses, color vision, tonometry, fundus examination or fundus photo, optical coherence tomography scan, and objective refraction.

**FIGURE 1:**
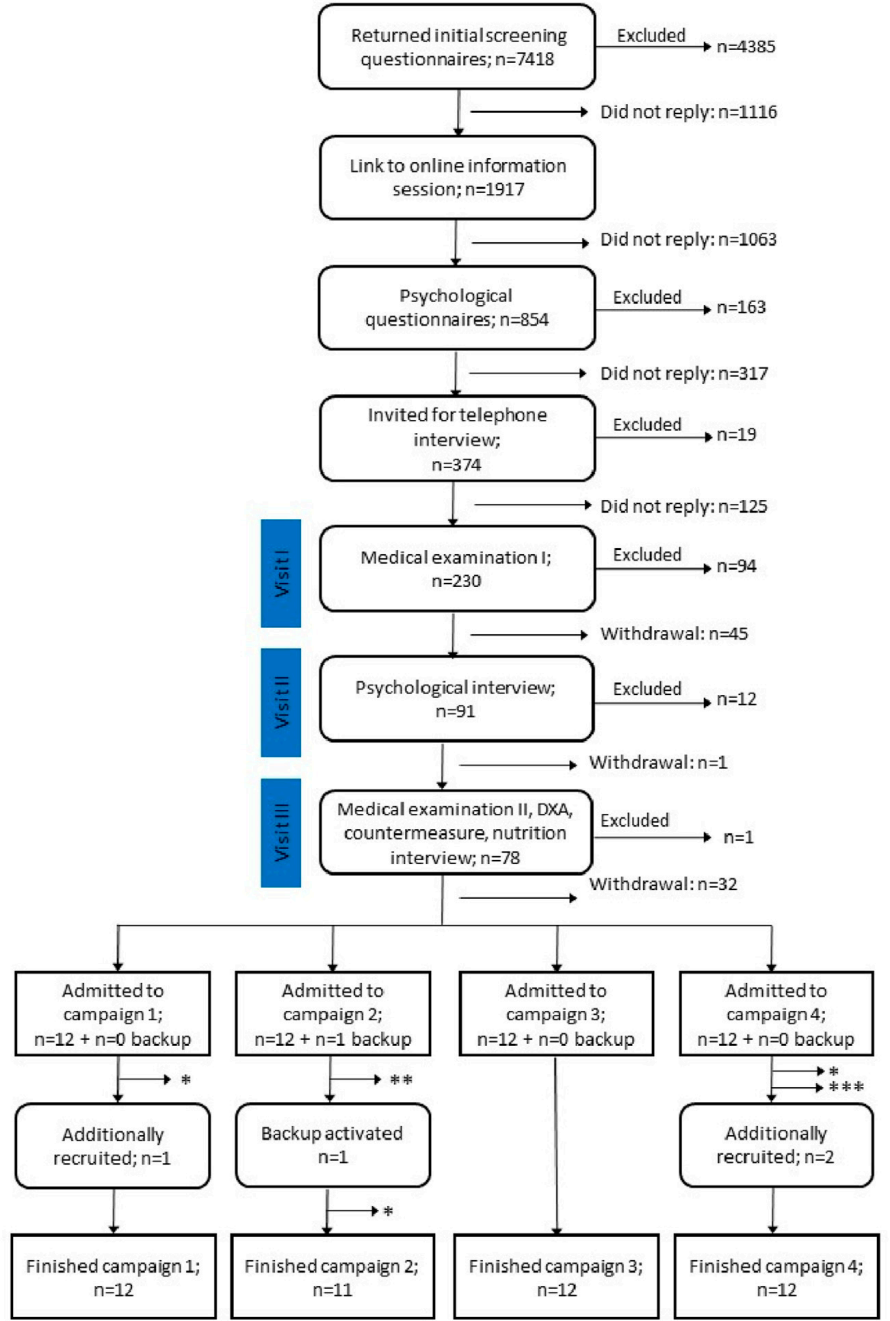
Flow diagram showing the recruitment process for the SANS CM study. Numbers are summed up for all four campaigns. From 7,418 applicants, 48 + 1 were admitted to the study. 5 participants dropped out of the study for reasons indicated by asterisks: * medical reason, ** violation of study regulations, *** personal reasons. After backup activation and additional recruitment, 47 participants completed the study.

Applicants passing initial medical screening were then invited to return to the DLR for an onsite assessment day (combining visit II and III), which started with an additional psychological questionnaire (temperament structure scales questionnaire) followed by an in-depth psychological interview. Applicants passing the interview had talks with the project lead, nutrition experts, subject coordination team, and countermeasure specialists, who also offered the opportunity to try out the LBNP-device and thigh cuffs. The assessment day was also the last opportunity to provide a criminal background clearance and proof of existing health insurance. Those who remained eligible and interested proceeded to further medical evaluations, which included bone density measurements of the lumbar spine and unilateral femoral head using dual energy-X-ray absorptiometry. In accordance with German radiation protection legislation, bone density assessment was scheduled as the concluding appointment. All steps of the recruitment process and the number of drop outs in between are illustrated in Figure 1. Certain tests such as HIV, hepatitis B/C, and tuberculosis screenings were repeated at enrollment.

### Study design

#### Location and outline

The SANS CM study took place at :envihab, a medical research facility of the DLR Institute of Aerospace Medicine in Cologne, Germany. The facility is equipped with 12 single-person bedrooms, multiple bathrooms (both for upright showering and for HDT showering using gurneys), a communal room with living and dining areas, multiple examination rooms, including a 3T PET-MRI (Biograph mMR, Siemens Healthineers, Munic, Germany), and a metabolic kitchen.

The study was divided into four campaigns, each accommodating 12 participants. The first campaign started in September 2021 and the fourth ended in July 2023. Irrespective of the campaign, all participants resided for 59 days continuously at :envihab, divided into three different phases: Phase 1: 15 days of baseline data collection (BDC), running from BDC-15 to BDC-1; Phase 2: 30 days of HDT bed rest, running from HDT1 to HDT30; Phase 3: 14 days of recovery (R), running from R+0 to R+13. In all three phases, participants were not allowed to leave :envihab, with the exception of three examinations at other sites. Participants had to return for follow-up examinations 90 and 540 days after finishing bed rest.

Group assignment was done via stratified randomization. First, the recruitment team identified participant pairs based on gender, anthropometrics, and age characteristics. Next, the codes of these participant pairs were handed to another person, who used a random generator (www.random.org) for assigning one participant per pair to one of the countermeasure groups (LBNP in campaign 1 + 2 and thigh cuffs in campaign 3 + 4). The other participant was automatically assigned to one of the control groups (upright seated in campaign 1 + 2 and no countermeasure in campaign 3 + 4). In campaigns 1 and 2, six participants per campaign were assigned to the lower body negative pressure group (LBNP; six men and six women) and six participants per campaign to the upright seated group (SEATED; six men and five women; see Figure 1 for details). Similarly, in campaign 3 and 4 we allocated participants to the exercise and venous constriction thigh cuff group (EX+CUFF; eight men and four women) or to the negative control group (CONTROL; seven men and five women). Table 1 provides participant demographics for each group. One-way ANOVA revealed no statistical difference between the four groups for age (p = 0.87), height (p = 0.87) and weight (p = 0.93).

**TABLE 1:** Demographic characteristics of all 47 participants. Results are sorted by group and sex and are represented as mean values ± one standard deviation. There was no statistical difference between the four groups for age (p = 0.8678), height (p = 0.8652) and weight (p = 0.9277).

	LBNP (n = 12)		EX+CUFF (n = 12)		SEATED (n = 11)		CONTROL (n = 12)		Total (n = 47)	
	Male	Female	Male	Female	Male	Female	Male	Female	Male	Female
N	6	6	8	4	6	5	7	5	27	20
Age [years]	32 ± 10	41 ± 11	35 ± 10	34 ± 8	34 ± 8	32 ± 4	37 ± 10	33 ± 6	34 ± 9	35 ± 8
Height [cm]	178 ± 5	166 ± 8	179 ± 7	166 ± 7	181 ± 5	168 ± 6	183 ± 9	164 ± 6	180 ± 7	166 ± 7
Weight [kg]	81 ± 12	66 ± 16	73 ± 10	64 ± 10	76 ± 7	66 ± 7	80 ± 14	61 ± 3	77 ± 11	65 ± 10

#### Standard bed rest conditions

**FIGURE 2:**
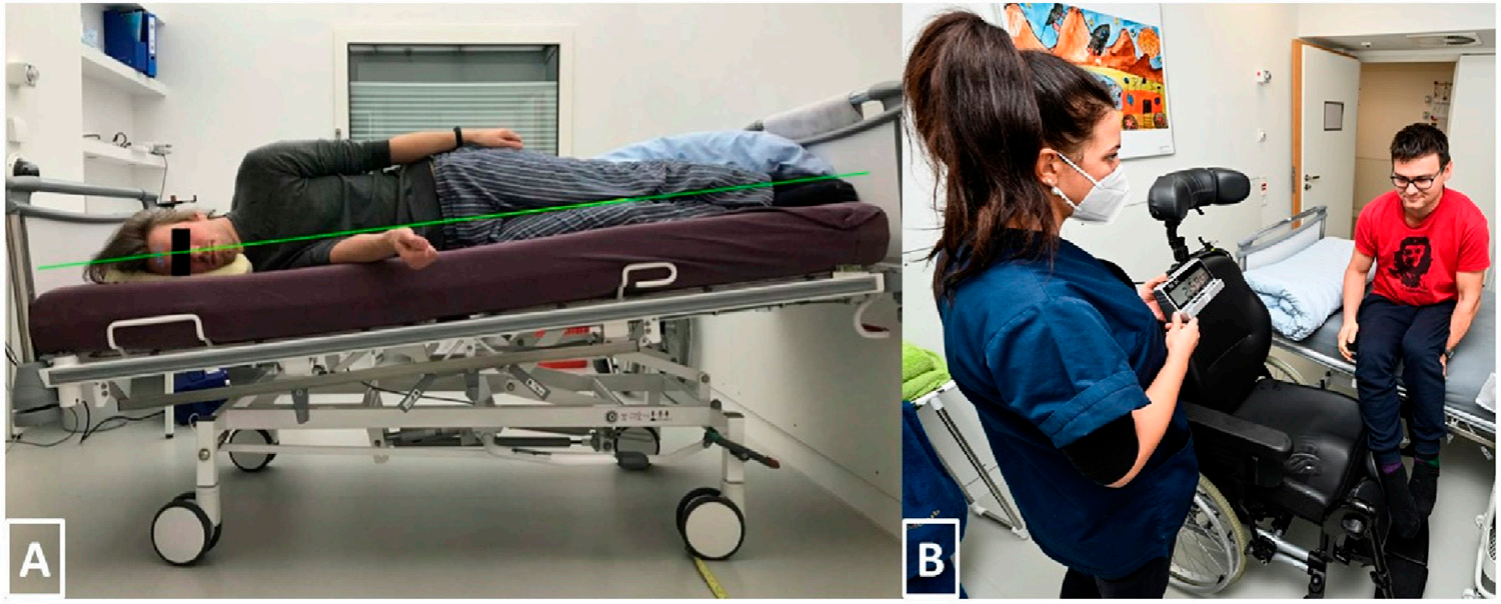
**(A)** Strict HDT bed rest. Only a side-pillow was allowed to assure that the whole body and head remained aligned with the −6° angle. **(B)** After 3 hours, participants of the SEATED-group had to switch from upright seated back to head down tilt. Due to the removable armrest, participants were able to do the transmission without having their feet touching the ground.

In order to model SANS-like findings, this study adopted the established strict HDT bed rest model, meaning that no pillow was allowed, except for side sleeping, to ensure the −6° angle was maintained from head to toe, as displayed in Figure 2A. Furthermore, this study adhered to the international guidelines recommended for HDT bed rest (Sundblad et al., 2014). Essential daily activities such as eating, washing, showering, bowel movement, as well as all leisure activities (e.g., reading books or watching television) were carried out in the 6° HDT position. Deviation from this position (such as sitting or standing) was not allowed during the bed rest phase, though participants were free to change their body position (prone, side, supine), given that one shoulder remained in contact with the mattress at all times. Beside these movements, static as well as dynamic muscle contractions were not permitted, but daily stretching of key muscle groups and joints was required. Physiotherapy was provided every other day to avoid muscle contractures, muscle stiffness, and back pain. The SANS CM study required that participants should be able to get 8 h of sleep. No daytime napping was allowed.

During all three phases, participant rooms were video monitored during the night. During the HDT phase, video monitoring was extended to 24/7. This procedure ascertained participant safety and assured compliance with the night time and bed rest directive. During urination or bowel movements, participants were allowed to cover the camera. There was no video surveillance in the shower. Participants signed a dedicated consent form for video surveillance. Monitoring the video feeds was only allowed for nursing staff and project team members. In case of suspicious results or observations that could hint towards protocol violations, the project team had the authority to check the recorded videos to safeguard study integrity.

#### Nutrition

Throughout the study, participants ingested a strictly controlled standardized diet, adapted to each participant, based on body weight and resting energy expenditure. Resting energy expenditure was measured via indirect calorimetry in the morning (fasting) of the first study day (BDC-14). In the ambulatory phase, physical activity level was set to 1.6 (equivalent to light physical activity) and during bed rest to 1.4 (equivalent to adults with a sedentary lifestyle) times the resting energy expenditure. To maintain body weight during bed rest within 3% of the one measured on HDT3, energy intake was individually adjusted by varying carbohydrate and fat intake. Energy intake was targeted to meet individual and international bed rest standards (Sundblad et al., 2014) as follows: protein intake was set at 1.2 g per kg body weight and day, fat intake range of 30%–35% of total energy intake, at least 30 g/day of dietary fiber were included in the diet and carbohydrates provided the remaining energy.

The standardized diet was developed and supervised by nutritionists and dieticians. Using PRODI software (Kluthe Prodi 7.1 Expert, Nutri-Science GmbH, Freiburg, Germany), 14 different menus were created and individually adapted to the needs of participants, as well as to study phase and day. These menus were repeated four times throughout the study and comprised three main meals and one snack. Timing of food intake was adapted to the countermeasure protocol and other study activities. All meals were prepared and weighed to the exact gram by trained staff in the :envihab metabolic kitchen. Thereby, the intake of vitamins, minerals, and other micronutrients was strictly controlled and standardized. To avoid vitamin D deficiency due to reduced sunlight exposure, 1,000 international units vitamin D were supplemented per day. The menus contained standard german food items. Caffeine, black tea, herbal tea, chocolate, alcohol or other metabolism stimulating drinks were prohibited. Participants had to follow the predetermined standardized diet; only food provided by the metabolic kitchen was permitted. Meals had to be eaten entirely and preferably within 30 min. The fluid intake was documented daily, but the volume could be adjusted upon request by the participants.

Table 3 contains the nutritional requirements and actual intake for all four SANS CM campaigns. Macronutrient, water, sodium, and calcium intake was checked daily. All other nutrients such as vitamins and minerals were assessed on a weekly basis.

#### Scientific Experiments

The SANS CM study was part of NASA’s Human Research Program to address the needs for human health and performance risk mitigation strategies to enable space exploration. Under the premise to initiate international collaboration for the most ethical, economical, efficient, and strategic use of available resources, proposals were either selected based on an independent standard scientific merit (peer) review, coordinated by the NASA Peer Review Service, or based on an internal merit review at the DLR Institute of Aerospace Medicine. As a result, five principal investigators from NASA (22 experiments) and seven principal investigators from DLR (45 experiments) were selected for the SANS CM study. Additional 19 experiments were done as part of the international standard measures which gives a total of 86 experiments. Because experiments were repeated several times, each participant completed 268 scientific sessions, resulting in a total of 12,596 scientific sessions throughout all four campaigns of the SANS CM study. This number does not include procedures like physiotherapy, daily vitals, daily countermeasures, daily questionnaires or isolated blood sampling. A table containing a streamlined overview of the experiments is part of the supplementary material.

#### Medical care

To ensure safety and wellbeing, medical care was available around the clock. Medical staff conducted daily ward rounds to monitor health status. All medications were monitored and recorded in the clinical report throughout the study. Careful attention was paid when administering medications to avoid potential confounding effects on experiments. Clinical laboratory parameters to assess medical safety were evaluated from blood and urine samples collected throughout the study (BDC-14, HDT10, R+1, R+10). Oral body temperature, heart rate and brachial blood pressure were measured on a daily basis immediately after the scheduled wake up. Every morning, following the first urination, body weight was assessed. Urine was collected daily for 24 h throughout all three study phases. Body height was recorded on study phase transition days (BDC-14, HDT1, R+0).

Before and after HDT bed rest, ocular, ear, nose and throat examinations were performed by DLR medical specialists as part of general healthcare. Additionally, participants had the opportunity to participate weekly in individual consultation sessions with an external and independent psychologist.

During the baseline data collection phase, a negative urinary pregnancy test based on the morning void was required before female participants were exposed to dual energy-X-ray absorptiometry or MRI. Following this phase, the participants were required to verbally state that they were not pregnant before commencing these tests.

### Countermeasures, tolerability and positive control

#### Lower body negative pressure

##### Requirements

As participants had to spend 6 h per day in the LBNP-device for 30 consecutive days, an important goal when developing the LBNP-device was to make it as comfortable as possible. Accordingly, the possibility to change body position, for example, rotating on ones’ side, during LBNP-sessions was an important requirement. For operational reasons, the 6 h were split into two sessions with 3 h LBNP in the morning and 3 h in the afternoon. In case of an interruption, e.g., for changing body position or having a toilet break, LBNP-time had to be stopped and added afterwards to achieve exactly 6 h LBNP per day. As we had six sessions running in parallel, LBNP-devices had to be constructed such that entering and leaving the device required as little time as possible while maintaining strict 6° HDT position. Another requirement was the possibility to observe participant’s legs to ensure that they do not continuously contract their muscles while being inside the LBNP. Each LBNP-device shall maintain a negative pressure of 25 mmHg within a range of ±2 mmHg. Pressure, temperature and humidity inside the chamber were to be monitored in real time and recorded to an external device. To document hemodynamic responses to LBNP, continuous finger blood pressure had to be recorded during every session.

##### Development

**FIGURE 3:**
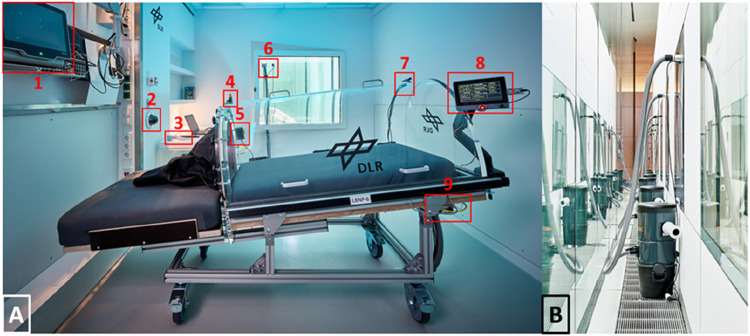
**(A)** Lower body negative pressure device (LBNP) inside a patient room. The following details are marked: 1) Finger blood pressure device 2) Video monitoring 3) Intercom 4) Valve 5) Emergency button 6) Passages for power supply and pressure tube 7) Sensors for pressure, temperature and humidity 8) Control panel 9) Automatic pressure control unit. **(B)** For minimizing the noise inside the room, pumps generating the negative pressure were situated outside.

**FIGURE 4:**
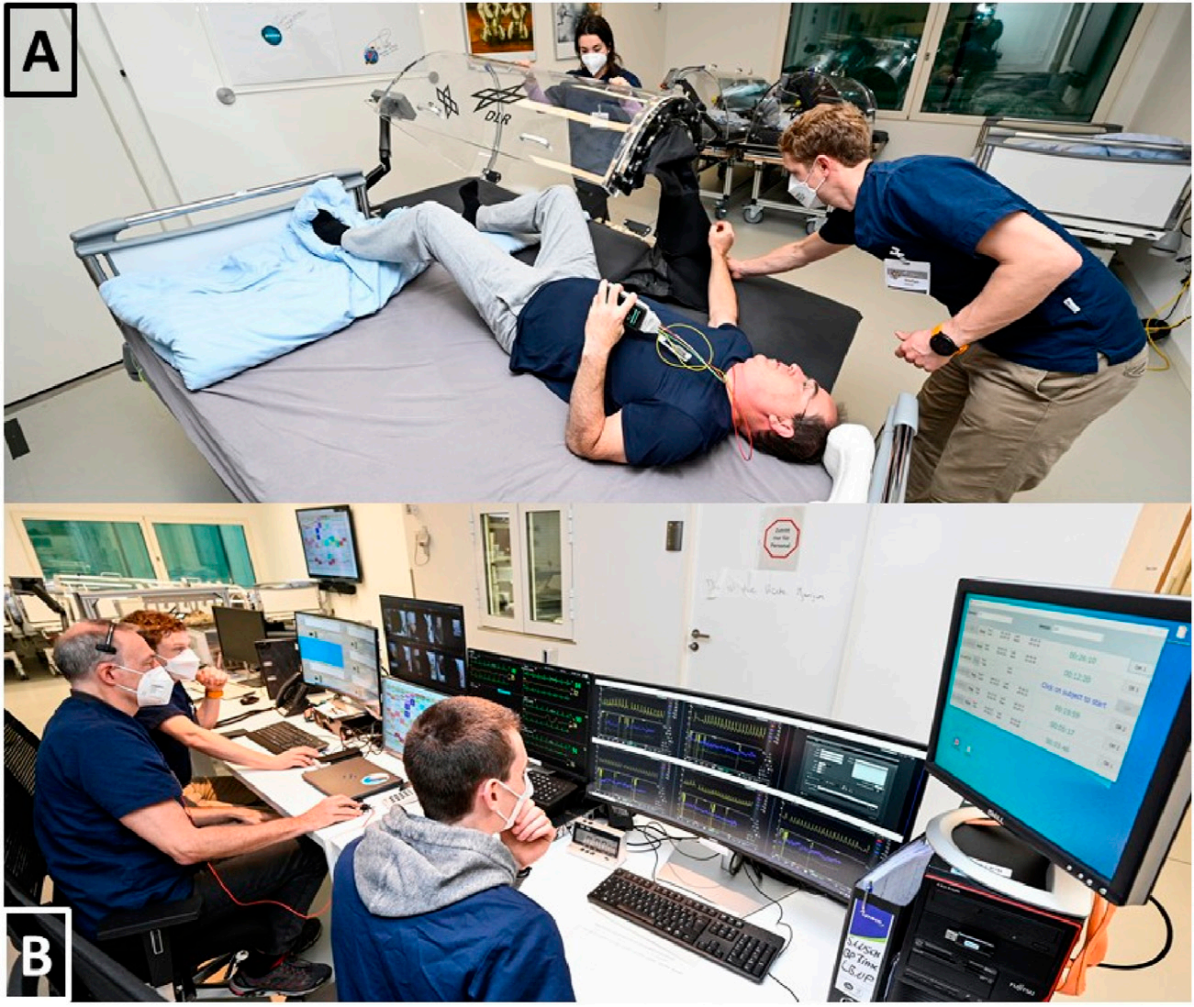
**(A)** The Neoprene-Skirt for sealing the LBNP-Chamber was equipped with an end-to-end zipper so the LBNP chamber could be opened to side. Thereby, participants were able to enter and exit the LBNP while maintaining the head down tilt directives. **(B)** The central station for controlling, monitoring and recording LBNP, ECG and finger blood pressure.

We addressed these requirements by building a LBNP-device that had a thick mattress and a chamber made out of acrylic glass (see Figure 3A). The chamber could be opened to the side so participants were able to roll from their bed into the LBNP-device or *vice versa* (see Figure 4A). Sealing the chamber at the level of the hip was done by using a neoprene skirt with an end-to-end zipper on the side where the chamber was opening. To avoid the neoprene skirt from getting sucked into the chamber, we added a wooden plate, individually tailored to hip dimensions, between the chamber and the skirt. We also added a leak valve to the chamber that could be opened by participants in case they felt hot inside the chamber. The LBNP-countermeasure was conducted inside participant rooms, to maintain their familiar environment and provide access to daily activities such as watching TV or playing video games. In each particpant room, we exchanged the window pane with acrylic glass equipped with passages for power supply and pressure tube. Thereby, the pumps creating negative pressure inside the LBNP-chambers were situated outside the rooms which minimized noise exposure. Pumps were regulated through a custom-built automatic pressure control unit, attached to the bottom of the LBNP-device. Both are displayed in Figures 3A, B. In principle, each of the 6 LBNP-devices could have been used standalone via the control panel connected to pressure control unit. To enhance participants’ privacy by closing the door to the corridor, the LBNP-devices were controlled remotely. Therefore, each pressure control unit was equipped with ethernet, and all LBNP-sessions were started, paused, and stopped at the central control station, which also recorded pressure, temperature and humidity inside the LBNP-chambers. Whenever a LBNP-session had to be paused, the remote-control software (LabVIEW 2016, National Instruments, Austin, Texas, United States) stopped and restarted the clock automatically and placed a predefined event marker. For continuous finger blood pressure recording, 6 Finapres NOVA (Finapres Medical Systems B.V., Enschede, the Netherlands) were used and also controlled remotely from the control station. The devices were set to switch from one finger cuff to a second every 30 min, followed by brachial blood pressure calibration. Medical monitoring was also done remotely using wireless electrocardiogram (IntelliVue MX40, Philips Medical Systems, Hamburg, Germany). In case of pre-syncopal symptoms, participants could either call the central control station via intercom or stop the LBNP-device via an emergency button. Within 720 LBNP sessions, both scenarios never occured. Finally, each participant room was equipped with a video camera, and the videos were displayed real-time at the central control station. The entire central control station is shown in Figure 4B. The LBNP-countermeasure hardware and control station with associated custom-built monitoring software were developed and constructed by the DLR.

##### Implementation

Before the start of the HDT phase, each participant completed a one-hour LBNP familiarization session. During HDT bed rest, LBNP-sessions in the morning started between 8:30 a.m. and 10:00 a.m. LBNP-sessions in the afternoon started between 2:00 p.m. and 3:30 p.m. Onset of each LBNP-session deliberately varied each day and the two daily sessions were separated by at least 1 h. Each session began by transporting participants to the central control station where they moved from bed to LBNP-device. After instrumentation for medical monitoring, LBNP-device and participant were transported back to their room. Next, both finger blood pressure cuffs and the brachial blood pressure cuff were donned. Then, the LBNP-device was connected to power supply, ethernet, and the pressure hose. After synchronizing LBNP and finger blood pressure recording, the LBNP-device was remotely started. The entire procedure from picking up the participant until the onset of LBNP took less then 15 min. LBNP-devices maintained −25 mmHg within ±0.2 mmHg and mean temperature and humidity were at 24°C and 36% respectively. If participants needed a toilet break during the LBNP-session, they moved out of the chamber onto a designated stretcher. Finger blood pressure measurement was stopped and the cuffs were removed. In general, elderly participants were more prone to toilet breaks than younger ones. After the initial adapation to HDT bed rest, the number of toilet breaks per participant (0-2 per session) remained constant. The entire procedure including restart of the LBNP-device took approxemetialy 5–15 min with male participants tending to be faster than female participants. Stopping the LBNP-session, switching back to the bed and returning to the participant room took less than 5 min.

#### Exercise and venous constrictive thigh cuffs

##### Requirements

The requirements stated that each cycling session should start with a 5-min warm-up at 50% of the target load followed by another 5-minute period at 75%. Target load was defined as the load in Watt that evokes 45% of the maximum oxygen consumption and shall be maintained for 45 min during each exercise session, followed by a 5-minute cool-down at 50% of the target load. Maximum oxygen consumption and target work load had to be determined by having participants cycle in the 6° HDT posture and increasing wattage 25 W each 1 min until volitional fatigue (VO_2_-peak-test). During the HDT bed rest period, steady-state metabolic rate was measured during the final 15 min of exercise at target workload on HDT6, HDT15, and HDT27. Participants had to cycle in the 6° HDT position at a cadence of 70–80 RPM. Heart rate and rating of perceived exertion had to be recorded every 15 min within in each session. After the cycling session, venous thigh constriction cuffs had to be tightened to 50 ± 5 mmHg within 15–30 min and had to be worn for 6 h continuously. Thigh cuff target pressure had to be monitored every 30 min. In contrast to the LBNP-group, the EX+CUFF countermeasure had to be applied for 6 days in a row, followed by one resting day.

##### Development

**FIGURE 5:**
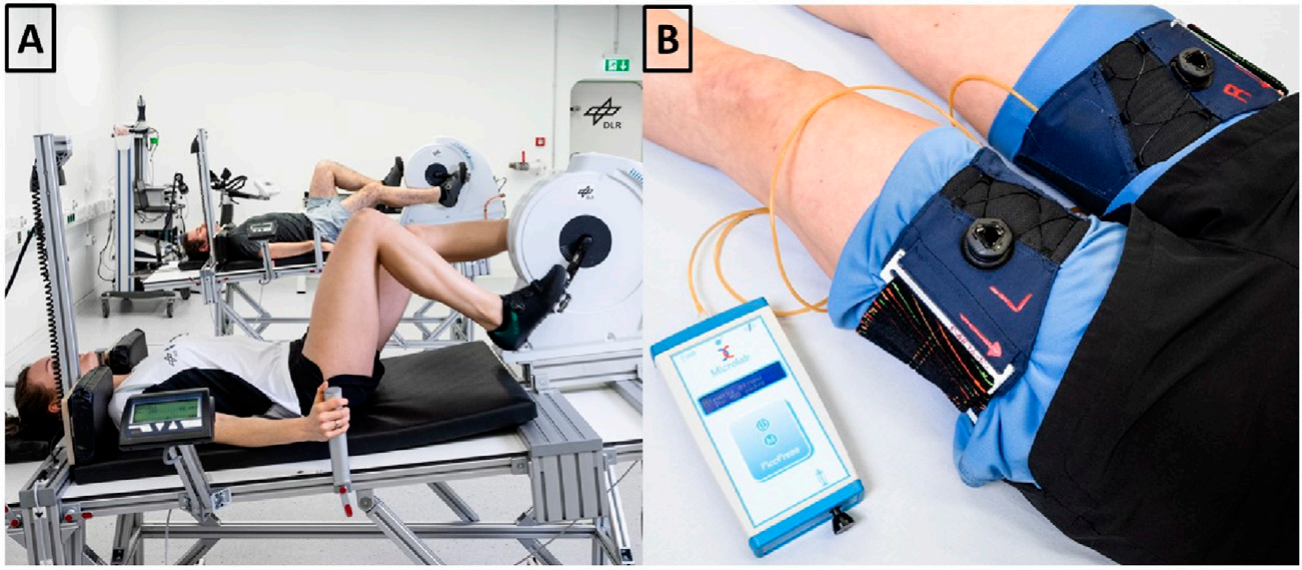
**(A)** We mounted recumbent bicycle ergometers on a customized 6° head down tilt stretcher. **(B)** Following cycling, venous constrictive thigh cuffs were donned and tightened to 50 mmHg, as verified with a digital manometer.

Exercise training was performed using two recumbent ergometers (Type 917900, Lode B.V., Groningen, Netherlands) mounted on identical custom-built stretchers in a 6° HDT position. Participants wore cycling shoes with clips (SPD system, Shimano, Sakai, Japan) to ensure efficient power transfer in this position. Shoulder padding and hand grips on the stretchers were adjustable. Displays showing the actual cadence were mounted within the field of vision. The entire setup (shown in Figure 5A) was built by the DLR.

Custom-built venous constrictive thigh cuffs (Clemson Textile Group at Clemson University) were ergonomically designed with stretchable fabric to fit snugly around the upper thigh, and similar to previously used versions (Marshall-Goebel et al., 2021; Balasubramanian et al., 2018). Velcro and a micro-adjustement knob allowed secure tightening. Cuff pressure was monitored using an air bladder placed in a pocket inside the cuff. The bladder was connected via plastic tube with a digital manometer (PicoPress, Microlab Elettronica Sas, Ponte San Nicolò, Italy). A washable fabric separated the skin from the cuff. NASA provided thigh cuffs, fabric and manometer (displayed in Figure 5B).

##### Implementation

Before the first cycling session, particpants tried out several thigh cuffs for determining appropriate cuff size. Then, thigh cuffs were inflated to 50 mmHg, and doppler flow measurement of the femoral vein was performed to verify sufficient venous constriction, defined as no detectable continuous blood flow. Simultaneously, participants were asked to perform several plantar flexions to ensure that 50 mmHg do not evoke an absolute venous occlusion. On BDC-13 and BDC-7, participants of the EX+CUFF-group completed the VO_2_-peak-tests on the 6° HDT cycle ergometer for determining the target work load. Heart rate, oxygen consumption, carbon dioxide production, ventilation rate and respiratory exchange ratio were continuously measured using a metabolic cart (Quark CPET, Cosmed, Rome, Italy) connected to the ergometer. Before onset of HDT bed rest, participants completed four familiarization sessions (BDC-12,-4,-2,-1) by cycling at the determined target load for 45 min. Following the familiarization exercise sessions, the participants practiced moving back to their beds and having the venous constrictive thigh cuff donned to 50 mmHg, but only wore them for 15 min.

During HDT bed rest, participants started cycling between 8:30 a.m. and 12:30 p.m. Starting time varied each day in a randomized manner or was chosen according to other experiments on that day. Cycling sessions were supervised by certified fitness coaches. Heart rate was obtained via ECG and 6-20 rating of perceived exertion via Borg-scale. Participants were allowed to use headphones while cycling. After the cycling session, participants were returned to their rooms for further cool-down, body hygiene (no shower), and changing clothes. Then, thigh cuffs were applied within 15–30 min while preventing formation of pinch points and fabric wrinkles underneath the cuff. Before the final pressure adjustment, participants flexed hip and knee twice to avoid pressure variations secondary to cuff movements. To avoid full venous blood flow stagnation for 6 h, participants were asked to repeat these movements plus plantar flexion every 30 min. Afterwards, we monitored and recorded cuff pressure as described in the requirements and made small adjustments in case of cuff pressure deviations. Participants were allowed to shower after the thigh cuffs were removed.

### Tolerability of mechanical countermeasures

Following every countermeasure session, participants had to rate their overall comfort. The scale ranged from 1 to 5, where one indicated very uncomfortable and five indicated very comfortable. An extended comfort questionnaire was completed on a weekly basis. Here, participants had to rate their overall comfort during the countermeasure sessions over the past 7 days. Besides several other questions regarding constructural aspects of the LBNP or cuffs, participants also had to state the number of hours that they would use the countermeasure if they have to do it on a daily basis for 6 months.

#### Positive control

##### Requirements

The LBNP and EX+CUFF countermeasures were intended to partially reverse the headward fluid shift, but it was unknown whether the magnitude or duration of countermeasure would be most critical. Therefore, we utilized another control group that was exposed to a complete fluid shift reversal. Participants of this SEATED-group should be sitting upright for 2 × 3 h to mirror the LBNP-group. Meanwhile, leg and foot activity should be kept to a minimum to maintain comparability with the other groups. The remaining 18 h should be spent in the strict HDT position.

##### Implementation

To enhance participants’ comfort and hygiene, we used a nursing wheelchair (Netti 4U CE Plus, Alu Rehab Aps, Ry, Denmark) equipped with a headrest and thick cushioning that was easy to clean. If presyncope had occurred, the nursing staff would have been able to easily move the wheelchair to a fully recumbent position. The chair had removable arm rests so participants could easily move from the bed into the wheelchair and *vice versa* (see Figure 2B). Participants were not allowed to move the chair and had to spend their upright period in front of a desk, inside their rooms, just like the LBNP group. Posture of the upper body and activity of the legs was monitored with a video camera positioned next to the desk. For toilet breaks, participants were moved onto another wheelchair equipped with a bed pan underneath. Timing of the intervention was matched to the LBNP group.

### Study endpoint and sample size justification

While this work describes the study design, safety and tolerability of the applied countermeasures, the overall goal of the study was to test countermeasure effectiveness in mitigating SANS-like symptoms. HDT bed rest can evoke several SANS-like changes in eye structure and the change in total retinal thickness, determined by optical coherence tomography, was set as the primary study endpoint (Macias et al., 2020; Laurie et al., 2020; Pardon et al., 2022). A simulation study was conducted to assess the statistical power for detecting an effect of either the LBNP- or the EX+CUFF-countermeasure, causing a reduction in total retinal thickness increase under a two-tailed approach defining statistical significance using p < 0.05. Data from a previous HDT bed rest study, where an increase in total retinal thickness of 54 ± 12.4 µm was observed on HDT30, were used to inform the simulation of the proposed study. With 12 participants per group, corresponding to the maximum bed occupancy in :envihab, the simulation calculated a power of 80% to detect a 15-micron improvement in the expected increase in total retinal thickness.

## Results

### Countermeasures

**FIGURE 6:**
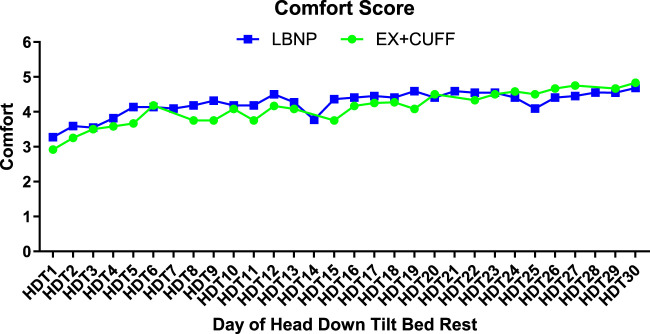
Comfort ratings of both SANS countermeasures throughout the HDT bed rest period. Results are represented as mean values for EX+CUFF- (dots) and LBNP-group (rectangles, mean of early and late session). A score of one is the lowest option and a score of five represents the highest comfort.

Next to the countermeasure effectiveness, tolerability is crucial for its implementation in future space missions. Comfort scores for both countermeasures are shown in Figure 6. The LBNP group had a slightly higher comfort score on HDT1 compared to the EX+CUFF group (3.3 vs. 2.9, respectively). Both groups showed increasing comfort ratings over the course of the HDT phase. At the end of bed rest, comfort ratings of both groups were almost identical (LBNP: 4.7 vs. EX+CUFF: 4.8). When we averaged comfort ratings over the whole study, the mean rating in the LBNP group was 3.9 in men and 4.4 in women (p = 0.1356, mixed effect model) and in the EX+CUFF group 4.2 in men and 3.9 in women (p = 0.1604). The mean comfort of the finger blood pressure measurement during the LBNP-sessions was rated as 2.6 (2.3 in men and 2.9 in women, p = 0.4408).

**FIGURE 7:**
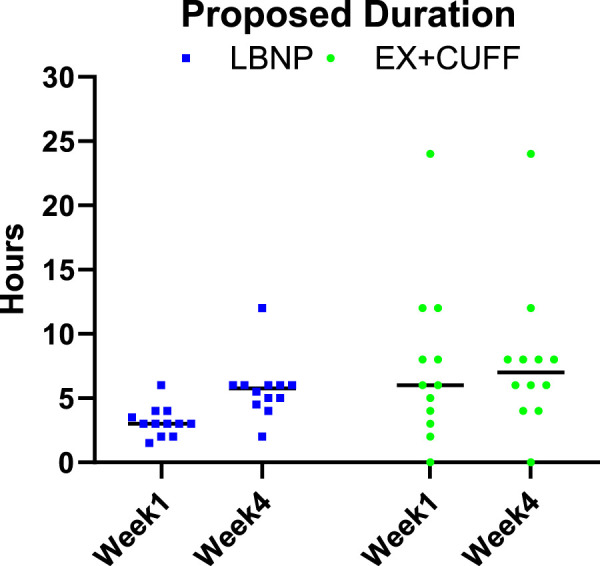
Participants were asked to propose reasonable durations of daily countermeasure application for future space missions based on their own experience. Results are represented as individual values and corresponding median for EX+CUFF- (dots) and LBNP-group (rectangles) in the early (week 1) and late (week 4) phase of head down tilt bed rest.

When we asked participants, which daily duration of countermeasure application they would find reasonable if being on a space mission, we observed differences over time and between both countermeasures. In the first week of HDT bed rest, participants proposed a duration of 6 h for thigh cuffs and 3 h for LBNP (see Figure 7). In the last week of HDT bed rest, participants found 7 h for thigh cuffs and 5.75 h for LBNP reasonable.

Out of 2,160 h scheduled LBNP-sessions, 4.5 h were not performed. One session had to be aborted after 1.5 h due to mental stress evoked by digestive problems. Another 3-hour session got canceled because a participant experienced vertigo unrelated to the LBNP-countermeasure. For the EX+CUFF-group, 1872 h were scheduled and 6 h are missing because one participant was sick one day. The participant did also not cycle that day. Another participant was also sick and passed over the cycling session, but did wear the thigh cuffs for 6 hours.

### Medical care

One participant experienced an adverse event associated with the EX+CUFF-countermeasure with several cutaneous hematomas on the left and right dorsal thigh varying in size. Hematomas occurred with horizontal stretching after wearing thigh cuffs for 15 min during the first familiarization session. The largest hematoma measured approximately 10 × 10 cm. There was no other reported trauma to the legs. As a result of the risk analysis, the participant switched groups with another participant because it was determined that her skin was uniquely sensitive to this level of skin contact pressure. However there were no other medical indications to explain this finding prior to selection and group assignment. Two pre-syncopal events were evoked by the upright seated countermeasure. The first event occurred on HDT5 after the participant was upright for 1 h and 28 min. The session continued after a 35-minute break in the HDT-position. The second event occurred in another participant on HDT10. No intervention was required because the participant recovered by itself. The same participant experienced severe unspecific rotary vertigo on HDT30 after being upright for 5 min. The session continued nominally after reclining the subject for 5 min. No adverse events were reported in the LBNP-countermeasure group.

Common HDT bed rest-related adverse medical conditions included vertigo (unspecific rotary vertigo and paroxysmal benign positional vertigo), headache, muscular pain, infections (e.g., ear inflammation), hoarseness, exanthema (e.g., skin irritations), presyncope (within the first hours after re-ambulation), mental stress, nausea, abdominal pain and tinnitus (see table 2).

**TABLE 2:** List of symptoms that occurred throughout the duration of the study, sorted by group. The table contains the absolut count of symptons and in parenthesis the number of participants reporting the symptoms. Please note that the presyncopes listed here did not occur during the application of the countermeasures.

	LBNP	EX+CUFF	SEATED	CONTROL
Vertigo	4 (2)	12 (4)	2 (1)	13 (8)
Headache	5 (3)	2 (1)	4 (4)	6 (4)
Muscular Pain	18 (8)	10 (5)	15 (8)	11 (5)
Infections	9 (4)	7 (3)	13 (7)	5 (3)
Hoarseness	1 (1)	4 (1)	0	1 (1)
Exanthema	7 (3)	2 (2)	6 (6)	4 (1)
Presyncope	1 (1)	1 (1)	1 (1)	2 (1)
Mental Stress	1 (1)	0	0	0
Nausea	4 (3)	0	1 (1)	3 (1)
Abdominal Pain	2 (2)	2 (1)	2 (2)	5 (2)
Tinnitus	1 (1)	0	2 (1)	4 (2)

In some participants, medically relevant conditions were identified during the study. In one participant, a thoracic spinal disc herniation was diagnosed during BDC-phase. The participant remained asymptomatic throughout the study and received physiotherapy. In another participant, a research-related brain MRI revealed aplasia of the A1 segment of the left anterior cerebral artery. A third participant presented a retinal hole with nearby vitro-retinal traction and no retinal detachment during BDC phase. Outpatient laser surgery was performed the next day to seal the hole. No further complications or influence on the study protocol were noted upon fundoscopy. However, for medical safety reasons, the participant switched from the LBNP-group to the SEATED-group before commencing HDT bed rest. In another participant, unspecific visual field defects without other ocular impairments or clinical deficits were detected during an ocular examination in the BDC-phase. Upon enrollment, one participant showed a slightly positive tuberculosis Quantiferon test (0.4 I.E, [<0.35 I.E., negative, >1 I.E., significantly positive]). The participant was promptly isolated, and subsequent chest X-ray and further laboratory testing for immunosuppressive diseases yielded negative results. Latent tuberculosis infection was diagnosed and repeat testing after study completion was recommended. During the tilt table experiment (BDC phase), one participant presented recurrent bigeminy, trigeminy, and isolated premature ventricular beats, occasionally occurring in clusters, with a tendency to increase while standing. The participant remained hemodynamically stable throughout the experiment and only experienced sporadic symptoms (slight “stumbling”). Premature ventricular beats had been previously diagnosed, but a cardiological workup was otherwise negative. The participant was closely monitored, received magnesium supplementation and potassium levels were maintained at a high normal level. One participant experienced an allergic reaction characterized by palpitations, convulsive syncope, and delayed reorientation during BDC following the application of proxymetacaine hydrochloride. The incident was rated as a serious adverse event, leading to the study exclusion and replacement by another subject, as the medication could not be replaced. During HDT bed rest, one participant presented with gastroparesis, featuring a mirrored appearance and a dilated stomach discovered incidentally in an MRI scan. Additional esophageal fluid retention was observed with no signs of reflux, pain, abnormal digestion, or acute abdomen, despite fasting for a day. An abdominal ultrasound revealed air-filled intestines and a fully filled, dilated bladder, indicating no signs of intestinal obstruction or urinary infection. To address the condition, nutrition was adjusted alongside laxative measures. Prokinetic medication was prescribed for several days. The abnormaility improved during the R+ phase with full recovery few weeks after the study. The strict standardized nutrition could not be fully maintained in this participant. A bulk of cerumen was removed in one participant during the BDC phase to prevent swelling and ear inflammation during HDT bed rest as a result of the fluid shift.

Safety PCRs for COVID-19 revealed positive results in two participants during one campaign. One had no clinical symptoms, the other had a mild sore throat and received symptomatic treatment. Specific precautions and measures to prevent further transmission including a rigorous isolation protocol of infected participants, education and training of staff and participants, remote care if possible, monitoring and adherence to the local health guidelines were immediately implemented. Antigen test results via nasal swab were negative after 1 day or 6 days, respectively. For the time of isolation, participants were suspended from scientific experiments but continued their participation in the countermeasure.

In general, supplementation was provided throughout the study for low levels of ferritin, vitamin D, and folic acid until they reached normal levels.

### Nutrition

Throughout all phases and campaigns, the mean protein intake was 14% of total energy and matched the targeted 1.2 g/kg body weight per day. Mean intake of carbohydrates (51%) and fat (32%) were also within in the targeted range. Following the recommendations for astronauts, mean sodium intake was kept under 2,300 mg per day. Mean fluid intake increased from HDT to R+ by 482 ml. More details are listed in Table 3. Daily energy intake was adjusted for cycling countermeasure (additional 250 kcal/training day) as well as reconditioning sessions (additional 0.1 x resting energy expenditure/day) by increasing fat and carbohydrates intake to compensate the energy loss. During bed rest, all body weights remained within ±3% of the body weight assessed on HDT3.

**TABLE 3:** Nutritional requirements and actual intake for the SANS CM study. Results are represented as mean values ±1 standard deviation from all 47 participants. Macronutrients, water, natrium and calcium were assessed on a daily basis. All other nutrients such as vitamins and minerals were assessed on a weekly basis.

Nutrients	BDC	HDT	R+	Nutritional requirements
	All groups	All groups	All groups	Bed rest studies
Total Energy (kcal/d)	2,559 ± 311	2,292 ± 307	2,641 ± 348	Maintain body weight
Protein (g/d)	86.45 ± 14.21	85.38 ± 14.80	85.92 ± 15.01	-
Protein (g/kgBW/d)	1.21 ± 0.07	1.19 ± 0.07	1.20 ± 0.06	1.2 g/kgBW/d
Protein (% of total energy)	13.84 ± 1.40	15.28 ± 1.77	13.32 ± 1.38	-
Total fat (g/d)	88.34 ± 11.62	79.25 ± 10.89	91.41 ± 12.75	-
Total fat (% of total energy)	32.06 ± 1.47	32.15 ± 1.51	32.14 ± 1.33	30%–35% of total energy
Monounsaturated fatty acids (g/d)	34.47 ± 6.22	30.10 ± 5.47	37.14 ± 6.94	-
Monounsaturated fatty acids (% of total energy)	12.50 ± 1.54	12.20 ± 1.52	13.04 ± 1.61	≥10% of total energy
Saturated fatty acids (g/d)	22.25 ± 4.27	19.53 ± 3.65	22.55 ± 4.51	-
Saturated fatty acids (% of total energy)	8.09 ± 1.28	7.93 ± 1.14	7.95 ± 1.29	≤10% of total energy
Polyunsaturated fatty acids (g/d)	27.32 ± 6.14	24.78 ± 6.09	27.47 ± 6.19	-
Polyunsaturated fatty acids (% of total energy)	9.92 ± 1.83	10.06 ± 2.13	9.66 ± 1.71	≥7% of total energy
Carbohydrates (g/d)	318.72 ± 39.90	276.47 ± 40.93	331.45 ± 44.39	-
Carbohydrates (% of total energy)	51.08 ± 2.11	49.44 ± 2.47	53.80 ± 57.10	50%–60% of total energy
Total Fiber (g/d)	38.33 ± 6.15	35.47 ± 4.98	39.97 ± 6.88	≥30 g/d
Fluid (ml/d)	3832.33 ± 495.53	3796.19 ± 573.40	4278.23 ± 671.25	-
Fluid (ml/kgBW/d)	54.08 ± 6.39	53.53 ± 6.89	60.56 ± 10.22	50 ml/kgBW/d
Calcium (mg/d)	1063.06 ± 50.47	1063.52 ± 57.84	1075.72 ± 53.61	1,000–1,200 mg/d
Chloride (mg/d)	3584.20 ± 227.94	3455.98 ± 263.27	3578.86 ± 285.85	-
Sodium (mg/d)	2273.85 ± 63.71	2264.15 ± 108.34	2263.75 ± 133.56	1,500–2,300 mg/d
Sodium (mmol/kgBW/d)	1.42 ± 0.24	1.41 ± 0.25	1.41 ± 0.25	-
Potassium (mg/d)	3897.35 ± 602.00	3648.83 ± 505.50	4031.09 ± 652.91	≥3.4 g/d (male), ≥2.6 m/d (female)
Fluoride (mg/d)	1740.79 ± 403.33	1659.26 ± 335.25	1769.70 ± 412.73	1.5–4 mg/d
Jodine (µg/d)	180.06 ± 98.25	168.55 ± 65.79	182.03 ± 98.93	≥150 μg/d
Copper (µg/d)	2105.51 ± 370.46	2067.26 ± 342.73	2251.14 ± 397.50	1,500–3,000 μg/d
Magnesium (mg/d)	455.91 ± 63.64	441.59 ± 66.09	481.09 ± 64.71	≥300 mg/d
Iron (mg/d)	27.46 ± 27.36	32.29 ± 30.51	35.72 ± 33.46	≥10 mg/d (male), ≥18 mg/d (female)
Phosphorus (mg/d)	1499.43 ± 207.56	1481.60 ± 199.34	1511.96 ± 210.23	700–1700 mg/d
Zinc (mg/d)	13.74 ± 3.57	13.13 ± 3.47	14.02 ± 3.29	12–15 mg/d
Vitamin B1, Thiamin (mg/d)	1.59 ± 0.27	1.51 ± 0.38	1.64 ± 0.27	≥1.5 mg/d
Vitamin B2, Riboflavin (mg/d)	1.92 ± 0.43	1.66 ± 0.40	1.93 ± 0.41	≥1.5 mg/d
Vitamin B3, Niacinequivalent (mg/d)	32220.21 ± 7125.06	30802.99 ± 5775.24	32876.89 ± 7108.20	≥20 mg/d
Vitamin B5, Pantothenic Acid (mg/d)	5.84 ± 1.06	5.79 ± 1.05	6.03 ± 1.19	≥5 mg/d
Vitamin B6 (mg/d)	2.38 ± 0.47	2.37 ± 0.56	2.45 ± 0.56	≥2 mg/d
Vitamin B7, Biotin (µg/d)	62.97 ± 35.38	52.23 ± 9.70	68.40 ± 35.10	≥30 μg/d
Viatmin B9, Folate (µg/d)	625.22 ± 198.01	601.08 ± 94.36	644.55 ± 202.26	≥400 μg/d
Vitamin B12, Cobalamin (µg/d)	6.71 ± 4.30	4.97 ± 3.22	6.04 ± 2.13	≥2 μg/d
Retinolequivalent (µg/d)	1578.35 ± 990.19	1842.16 ± 985.82	1565.39 ± 887.38	≥1,000 μg/d
Vitamin A Retinol (mg/d)	0.53 ± 0.35	0.51 ± 0.33	0.55 ± 0.35	-
Vitamin C (mg/d)	222.69 ± 62.58	215.43 ± 103.21	232.57 ± 63.36	≥100 mg/d
Vitamin D (µg/d), (diet + supplementation)	35.90 ± 12.93	33.06 ± 10.64	30.27 ± 4.61	Supplementation 1000 IU/d (25 μg/d)
Vitamin E (mg/d)	25.68 ± 7.13	26.20 ± 6.93	26.98 ± 7.35	≥15 mg/d
Vitamin K (µg/d)	218.96 ± 153.88	320.86 ± 195.35	229.65 ± 163.63	≥80 μg/d

## Discussion

The aim of this work is to introduce the design of the SANS CM study with a particular focus on countermeasure implementation and tolerability, in advance of additional manuscripts that will describe predetermined outcome measures. Previous studies showed that these countermeasures are well tolerated over shorter periods (Marshall-Goebel et al., 2021; Balasubramanian et al., 2018; Petersen et al., 2019; Lawley et al., 2020; Kermorgant et al., 2021), however, the question regarding their long-term tolerability has so far remained unanswered. The SANS CM study tested these countermeasures in the setting of a highly controlled, strict HDT bed rest study. The findings will have implications for conduct of future HDT bed rest studies and potential applications of such countermeasures during spaceflight.

With very few exceptions mainly related to medical reasons, strict HDT bed rest and standardization measures, particularly macro- and micronutrient ingestion, were rigorously implemented in the SANS CM study. Therefore, physiological responses to strict HDT bed rest and countermeasure tolerability, safety, and efficacy can be evaluated in the absence of potentially confounding variables. The very small number (<1%) of partially or fully missing countermeasure sessions is another strength and comparable to our 60-day HDT bed rest study, where participants were exposed to artificial gravity via short arm centrifugation (Frett et al., 2020). Unlike earlier studies, this study included a negative and a positive control group, which makes it possible to better gauge the benefit of each countermeasure on SANS.

With daily use, participants became more comfortable with their countermeasures, resulting in high ratings consistent with good tolerability for both countermeasures by the end of the study. These data highlight that habituation over several countermeasure sessions leads to improved comfort, and there is not a worsening of comfort. Possibly, tolerability ratings could also be affected by habituation to HDT bed rest, which usually lasts 3–5 days. The aspect of habituation seems to be confirmed by the proposed durations for both countermeasures, which increased from week one to week 4. The same questionnaire was also applied in a recent study, testing LBNP and venous thigh constriction for 45 min in a crossover design (Marshall-Goebel et al., 2021). Participants rated comfort at 3.3 for LBNP and at 4.0 for venous thigh constriction and suggested a future duration of approximately 2 h for LBNP and 4.4 h for venous thigh constriction. Given the small sample size of our study and the previous study, a clear conclusions cannot be ascertained for either countermeasure.

Both countermeasures, venous thigh constriction and LBNP, could pose specific medical risks. In our study, we did not observe clinically relevant countermeasure-related adverse events except one case of superficial hematomas in the EX+CUFF-group. The main risk of longer-term LBNP is syncope due to the hemodynamic stress imposed by the intervention. In this study, we used LBNP 6 h per day for 30 days consecutively and observed no presyncopal signs. In contrast, applying 1G at the center of mass for 30 min daily via shortarm centrifuge led to nine premature terminations of the countermeasure in a 60 days HDT bed rest study (Frett et al., 2020). Notably, the hemodynamic stress of LBNP in our study is less than that elicited by standing on Earth. However, individualized LBNP dosing, hemodynamic monitoring, and automated safety switch off could prove useful. Moreover, shorter-term LBNP has been safely applied in space for many years. Perhaps, the main concern when applying longer duration venous constriction thigh cuffs is venous thrombosis, which is a recently recognized health risk in space (Marshall-Goebel et al., 2019). Devices that restrict venous leg outflow have previously been applied in space without complications. However, if this countermeausure is further developed, additional medical monitoring would be warranted.

From a technical and operational point of view, venous thigh constriction may have certain advantages. For example, venous constrictive thigh cuffs have a high technical readiness level and are currently being investigated on the International Space Station. LBNP hardware suitable for longer term application in space has to be developed. From an operational point of view, venous thigh constriction is easier to apply. Moreover, compared to LBNP, venous thigh constriction requires less bulky equipment and could be implemented without electrical supply. This aspect is another advantage with respect to smaller spacecrafts and stations designed for future missions going beyond low earth orbit (Scott et al., 2019).

The SANS CM study has important limitations. While HDT bed rest is an established model to replicate various physiological effects of microgravity, the model cannot fully replicate real space conditions. Furthermore, HDT bed rest studies are not conducted in astronauts and selection criteria are not identical in study participants and in astronauts. For example, detailed thrombophilia screening, which we applied for safety reasons, could be considered genetic discrimination in an occupational setting. Another limitation is the rather small number of participants. We are pleased that our study included women and men, which reflects the current astronaut workforce. While we did not observe major differences in comfort ratings, our study was not sufficiently powered to quantify potential sex differences in countermeasure tolerability and safety. Previous studies suggested that women and men may exhibit differences in pain responses (Failla et al., 2024). Although testing countermeasures in HDT bed rest studies is less costly than similar studies in space, such studies are still expensive and labour intense, which limits the number of participants. Rigorous standardization is an important measure to reduce background noise and to permit meaningful analyses of HDT bed rest studies.

In summary, the SANS CM study compared two potential SANS countermeasures, namely, LBNP and venous thigh constriction following aerobic cycle ergometry exercise, against negative and positve control groups. We conclude that both countermeasures were well tolerated. However, additional studies may be required to exclude rare adverse health effects. Potential risks and practicability of these countermeasures will have to be weighed against health benefits revealed in various sophisticated investigations conducted as part of the SANS CM study. We hope that this study paves the way for successful SANS countermeasures, which remains an unmet health challenge in space.

## Data Availability

The raw data supporting the conclusions of this article will be made available by the authors, without undue reservation.

## References

[B1] Ahmed S. S. Goswami N. Sirek A. Green D. A. Winnard A. Fiebig L. (2024). Systematic review of the effectiveness of standalone passive countermeasures on microgravity-induced physiologic deconditioning. NPJ Microgravity 10, 48. 10.1038/s41526-024-00389-1 38664498 PMC11045828

[B2] Balasubramanian S. Tepelus T. Stenger M. B. Lee S. M. Laurie S. S. Liu J. H. (2018). Thigh cuffs as a countermeasure for ocular changes in simulated weightlessness. Ophthalmology 125, 459–460. 10.1016/j.ophtha.2017.10.023 29153458

[B3] Clément G. Rittweger J. Nitsche A. Doering W. Frings-Meuthen P. Hand O. (2022b). Assessing the effects of artificial gravity in an analog of long-duration spaceflight: the protocol and implementation of the AGBRESA bed rest study. Front. physiology 13, 976926. 10.3389/fphys.2022.976926 PMC9492851 36160844

[B4] Clément G. R. Crucian B. E. Downs M. Krieger S. Laurie S. S. Lee S. M. (2022a). International standard measures during the VaPER bed rest study. Acta Astronaut. 190, 208–217. 10.1016/j.actaastro.2021.10.017

[B5] Failla M. D. Beach P. A. Atalla S. Dietrich M. S. Bruehl S. Cowan R. L. (2024). Gender differences in pain threshold, unpleasantness, and descending pain modulatory activation across the adult life span: a cross sectional study. J. Pain 25, 1059–1069. 10.1016/j.jpain.2023.10.027 37956742 PMC10960699

[B6] Frett T. Green D. A. Mulder E. Noppe A. Arz M. Pustowalow W. (2020). Tolerability of daily intermittent or continuous short-arm centrifugation during 60-day 6o head down bed rest (AGBRESA study). PLoS One 15, e0239228. 10.1371/journal.pone.0239228 32946482 PMC7500599

[B7] Goswami N. Blaber A. P. Hinghofer-Szalkay H. Convertino V. A. (2019). Lower body negative pressure: physiological effects, applications, and implementation. Physiol. Rev. 99, 807–851. 10.1152/physrev.00006.2018 30540225

[B8] Kermorgant M. Sadegh A. Geeraerts T. Varenne F. Liberto J. Roubelat F.-P. (2021). Effects of venoconstrictive thigh cuffs on dry immersion-induced ophthalmological changes. Front. physiology 12, 692361. 10.3389/fphys.2021.692361 PMC8317025 34335300

[B9] Laurie S. S. Greenwald S. H. Marshall‐Goebel K. Pardon L. P. Gupta A. Lee S. M. (2021). Optic disc edema and chorioretinal folds develop during strict 6° head‐down tilt bed rest with or without artificial gravity. Physiol. Rep. 9, e14977. 10.14814/phy2.14977 34355874 PMC8343460

[B10] Laurie S. S. Lee S. M. Macias B. R. Patel N. Stern C. Young M. (2020). Optic disc edema and choroidal engorgement in astronauts during spaceflight and individuals exposed to bed rest. JAMA Ophthalmol. 138, 165–172. 10.1001/jamaophthalmol.2019.5261 31876939 PMC6990717

[B11] Laurie S. S. Macias B. R. Dunn J. T. Young M. Stern C. Lee S. M. C. (2019). Optic disc edema after 30 Days of strict head-down tilt bed rest. Ophthalmology 126, 467–468. 10.1016/j.ophtha.2018.09.042 30308219

[B12] Lawley J. S. Babu G. Janssen S. L. Petersen L. G. Hearon Jr C. M. Dias K. A. (2020). Daily generation of a footward fluid shift attenuates ocular changes associated with head-down tilt bed rest. J. Appl. physiology 129, 1220–1231. 10.1152/japplphysiol.00250.2020 32940563

[B13] Macias B. R. Patel N. B. Gibson C. R. Samuels B. C. Laurie S. S. Otto C. (2020). Association of long-duration spaceflight with anterior and posterior ocular structure changes in astronauts and their recovery. JAMA Ophthalmol. 138, 553–559. 10.1001/jamaophthalmol.2020.0673 32239198 PMC7118682

[B14] Mader T. H. Gibson C. R. Pass A. F. Kramer L. A. Lee A. G. Fogarty J. (2011). Optic disc edema, globe flattening, choroidal folds, and hyperopic shifts observed in astronauts after long-duration space flight. Ophthalmology 118, 2058–2069. 10.1016/j.ophtha.2011.06.021 21849212

[B15] Marshall-Goebel K. Laurie S. S. Alferova I. V. Arbeille P. Auñón-Chancellor S. M. Ebert D. J. (2019). Assessment of jugular venous blood flow stasis and thrombosis during spaceflight. JAMA Netw. Open 2, e1915011. 10.1001/jamanetworkopen.2019.15011 31722025 PMC6902784

[B16] Marshall-Goebel K. Macias B. R. Laurie S. S. Lee S. M. Ebert D. J. Kemp D. T. (2021). Mechanical countermeasures to headward fluid shifts. J. Appl. physiology 130, 1766–1777. 10.1152/japplphysiol.00863.2020 33856253

[B17] McCord J. L. Beasley J. M. Halliwill J. R. (2006). H2-receptor-mediated vasodilation contributes to postexercise hypotension. J. Appl. Physiology 100, 67–75. 10.1152/japplphysiol.00959.2005 16141376

[B18] Pardon L. P. Greenwald S. H. Ferguson C. R. Patel N. B. Young M. Laurie S. S. (2022). Identification of factors associated with the development of optic disc edema during spaceflight. JAMA Ophthalmol. 140, 1193–1200. 10.1001/jamaophthalmol.2022.4396 36301519 PMC9614681

[B19] Petersen L. G. Lawley J. S. Lilja‐Cyron A. Petersen J. C. Howden E. J. Sarma S. (2019). Lower body negative pressure to safely reduce intracranial pressure. J. physiology 597, 237–248. 10.1113/JP276557 PMC6312426 30286250

[B20] Scott J. P. R. Weber T. Green D. A. (2019). Introduction to the frontiers research topic: optimization of exercise countermeasures for human space flight - lessons from terrestrial physiology and operational considerations. Front. physiology 10, 173. 10.3389/fphys.2019.00173 PMC6416179 30899226

[B21] Sun X. Q. Yao Y. J. Wu X. Y. Jiang S. Z. Jiang C. L. Cao X. S. (2002). Effect of lower body negative pressure against orthostatic intolerance induced by 21 days head-down tilt bed rest. Aviat. space, Environ. Med. 73, 335–340. 11952053

[B22] Sundblad P. Orlov O. Larina I. Cromwell R. (2014). Guidelines for standardization of bed rest studies of bed rest studies. Paris: International Academy of Astronautics. 10.1152/japplphysiol.00089.2016 26917693

